# Astrocytoma with myelin oligodendrocyte glycoprotein antibody associated encephalomyelitis: A case report

**DOI:** 10.1097/MD.0000000000031003

**Published:** 2022-10-07

**Authors:** Guanghong Zhong, Jia Zhang, Xi Liu, Shaoming Yang, Hongli Gu

**Affiliations:** a Heyuan People’s Hospital, Heyuan, Guangdong, China.

**Keywords:** astrocytoma, encephalomyelitis, MOG-IgG, MRI, serum

## Abstract

Demyelination is similar with malignancy in clinical symptoms. Magnetic resonance imaging (MRI) is an important auxiliary examination in the diagnosis of demyelinating diseases and malignancy. Since MRI and symptoms can be difficult to distinguish demyelination from malignancy, other auxiliary examinations, such as demyelinating disease-specific antibodies, play an important role in distinguishing them. Previous studies have reported demyelinating disease-specific antibodies in patients with malignancy. What’s more, it is more difficult to confirm the diagnosis when the malignant tumor co-occurs with demyelinating diseases, which has never been reported in previous studies. We report the diagnosis of myelin oligodendrocyte glycoprotein antibody associated encephalomyelitis (MOG-EM) in a patient who had astrocytoma for several years.

**Case presentation:**

Patient’s concerns and diagnoses: our case report records a 49-year-old woman with astrocytoma for more than 4 years, who recently developed the symptoms of MOG-EM, including dizziness, vomiting, and vision loss. This astrocytoma patient was diagnosed with MOG-EM according to comprehensive evidence, including MRI, visual evoked potential (VEP), serum myelin oligodendrocyte glycoprotein antibody (MOG-IgG), and therapeutic effect. Interventions and outcomes: this patient was diagnosed with astrocytoma by surgical biopsy 4 years earlier. This patient has been treated with tumor resection, postoperative radiation treatment and chemotherapy. After treatment, the patient was left with right limb weakness while other symptoms were improved. Recently, the intravenous steroid agent was used to treat this patient after being diagnosed with MOG-EM. Dizziness, vomiting, and vision loss have gone into remission. This patient did not relapse in 7 months after discharge. This patient is still being followed up at the outpatient clinic. And the patient will next be treated with azathioprine.

**Conclusions:**

In previous studies, polyclonal antibody has been found in cancer patients, such as aquaporin-4 and MOG-IgG in astrocytoma patients. But the case of our study finds that astrocytoma can coexist with MOG-EM. Therefore, MOG-EM should not be excluded easily in astrocytoma patients when the relative antibody of encephalomyelitis is positive. What’s more, it reminds us that the pathogenesis of MOG-EM might be related to astrocytoma.

## 1. Introduction

Myelin oligodendrocyte glycoprotein antibody associated encephalomyelitis (MOG-EM) is an inflammatory demyelinating disease of the central nervous system. In the earliest studies, MOG-EM was often misdiagnosed as multiple sclerosis (MS) or classified as one type of neuromyelitis optic spectrum disorders (NMOSDs), which is oligodendrocyte glycoprotein antibody positive. MOG-EM is similar with NMOSDs which consists of several syndromes, including optic neuritis, myelitis, acute brainstem syndrome, and acute disseminated encephalomyelitis. However, with the further exploration of demyelinating diseases, a lot of studies found that immunopathogenesis of MOG-EM is different from aquaporin-4 antibody positive NMOSDs so that more and more experts classified MOG-EM as a separate disease category.^[1]^ The differences are as follows^[2–4]^: with the exploration of previous immunological studies, MOG-IgG can directly cause the occurrence of demyelinating diseases; MOG-EM does not damage astrocytes and lacks aquaporin-4 (AQP4) -IgG-positive; MOG-EM and NMOSDs have different histopathological features. The underlying pathogenesis might be relative to humoral immunity and antigen-antibody response which is still unclear. The diagnosis of MOG-EM is based on the evidence, including the symptoms, serological antibody, and magnetic resonance imaging (MRI).^[1,5]^

Astrocytoma was defined as tumor which presents character of astrocyte differentiation. Astrocytoma accounts for about 30% of all intracranial tumors and more than 78% of gliomas. Astrocytoma occurs mostly in frontal lobe, temporal lobe, thalamus, brain stem and cerebellar hemispheres.^[6]^ Astrocytoma can present similar symptoms with MOG-EM. And the presentation of MRI in astrocytoma makes it difficult to differentiate astrocytoma from MOG-EM.^[7]^ It is difficult to diagnose between demyelination and malignancy with MRI and symptoms.

Our case reports a 49-year-old woman with medical history of astrocytoma (WHO grade II, isocitrate dehydrogenase 1) for more than 4 years who was recently diagnosed with MOG-EM. This patient was diagnosed with astrocytoma by surgical biopsy, and treated with tumor resection, postoperative radiation treatment and chemotherapy (temozolomide) 4 years earlier. She developed the symptoms of MOG-EM, including dizziness, vomiting, and vision loss 2 months earlier. The clinical symptoms and MRI are unable to provide sufficient evidence for us to differentiate astrocytoma recurrence from MOG-EM. However, serum MOG-antibody, visual evoked potential (VEP), and therapeutic effect finally proved the diagnosis of MOG-EM.

## 2. Case presentation

### 2.1. Past medical history

Presentation: This patient is a 49-year-old woman who had received treatment with main complaint of right limb weakness and headache in hospital for the first time on August 16, 2017. The patient’s right limb weakness continued to worsen one month before admission, and her headache occurred a week before admission.

**Figure 1.**
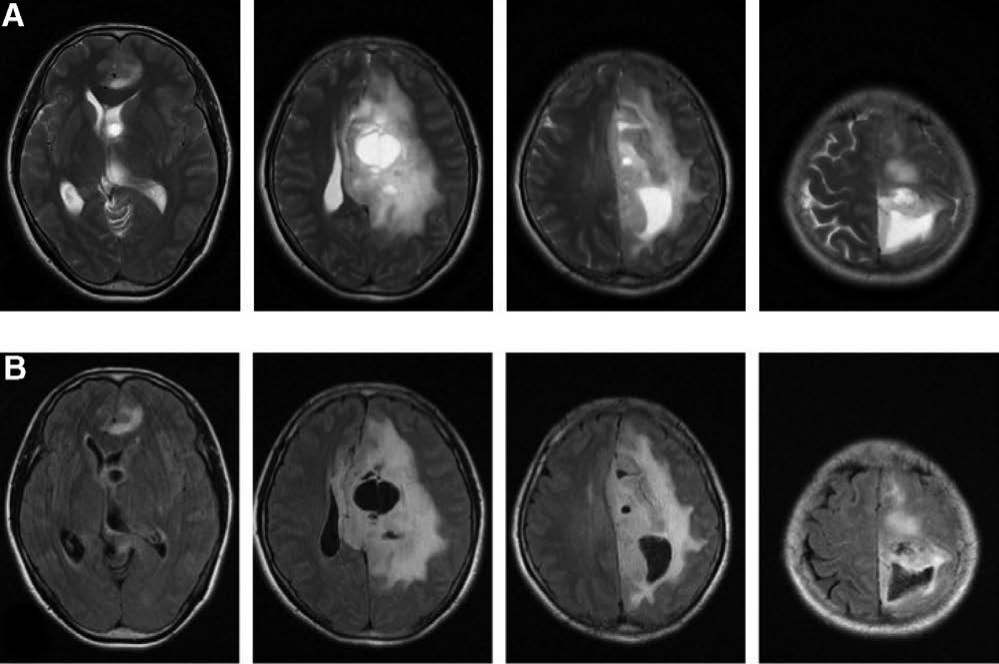
The MRI of pre-operation. (1) T2 (A) and T2 flair (B). (2) Left intracranial space-occupying lesion in frontal lobe, parietal lobe, and thalamus. (3) Midline displacement of cerebral hemispheres and subfalcine hernia. MRI = magnetic resonance imaging.

**Figure 2.**
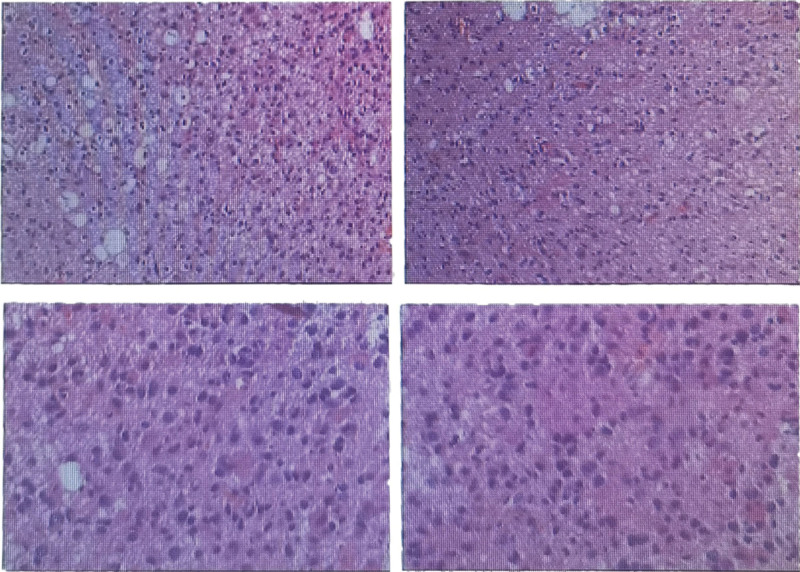
The surgical biopsy (hematoxylin and eosin). (1) Tumor cells were large round or polytonality which were arranged in honeycomb or lumpy form. (2) The nuclei are round, center, and hyperchromatic.

Physical examination, auxiliary examination, diagnosis, and treatment: Neurological examination presented right Hemiplegia (Muscle strength: level 3), and no another abnormal neurological sign. The MRI of pre-operation was shown in Figure 1. T2 and T2 flair presented left intracranial space-occupying lesion in frontal lobe, parietal lobe, and thalamus. And the MRI presented midline displacement of cerebral hemispheres and subfalcine hernia. This patient received the treatment of tumor resection in left frontal parietal lobe and thalamus. Due to the tumor lesion in brain functional region, complete tumor resection would cause significant damage to the patient so that this patient finally received partial tumor resection. The surgical biopsy was shown in Figure 2. Tumor cells were large round or polytonality which was arranged in honeycomb or lumpy form. The nuclei are round, center, and hyperchromatic. Immunohistochemistry presented positive isocitrate dehydrogenase 1 in this tissue. The surgical biopsy of intracranial space-occupying lesion was in accord with astrocytoma (WHO grade II, isocitrate dehydrogenase 1). The patient received radiotherapy and chemotherapy of temozolomide after surgery. After treatment, the patient was left with right limb weakness while another symptom was improved.

### 2.2. History of present illness

Presentation: This patient developed dizziness, vomiting, and vision loss before one month of this admission (November 6, 2021). The physical examination outcomes are similar to that of the first-time treatment except vision loss.

**Figure 3.**
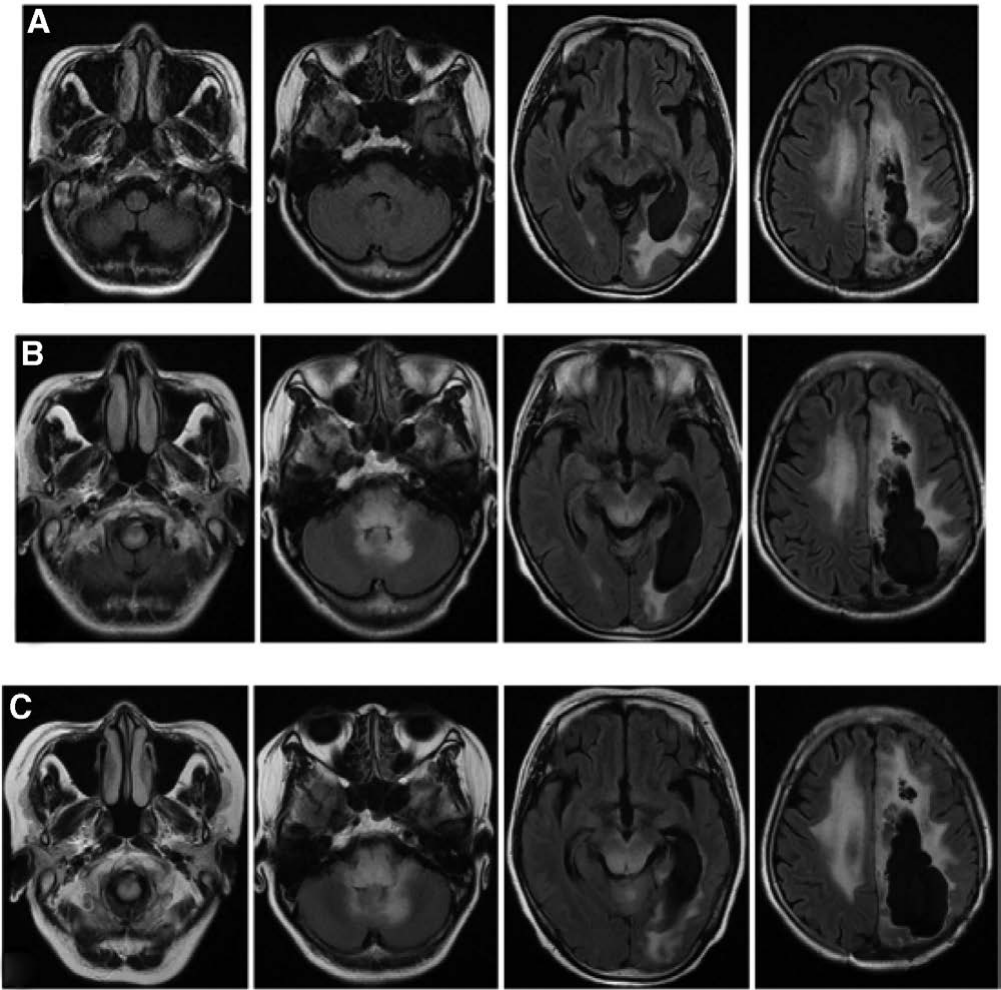
The T2 flair sequence of MRI. (1) Post-operation (A), this admission (B) and follow-up after discharge (C); (2) compared with post-operation, the brain stem and cerebellum developed diffuse hyperintensity at this admission; (3) compared with this admission, the brain stem and cerebellum developed more diffuse hyperintensity during follow-up.

**Figure 4.**
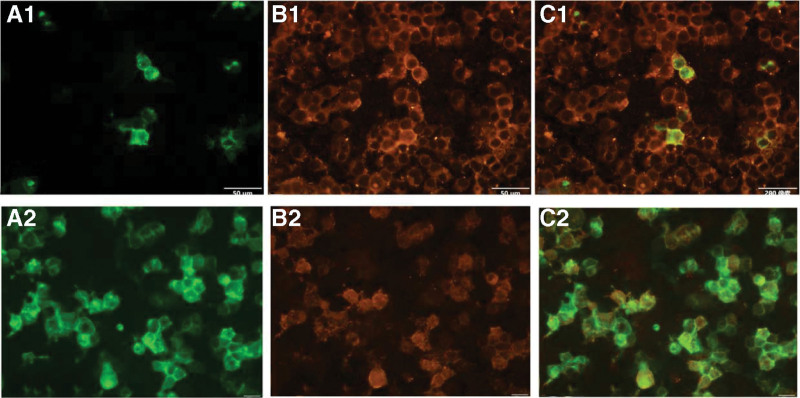
The outcome of serum MOG-IgG. MOG-IgG detection: (A) fluorescent of MOG. (B) Fluorescent of MOG-IgG. (C) Fluorescent of MOG overlaps with fluorescent of MOG-IgG. 1 = outcome of this admission. 2 = outcome of the reexamination. MOG-IgG = myelin oligodendrocyte glycoprotein antibody.

**Figure 5.**
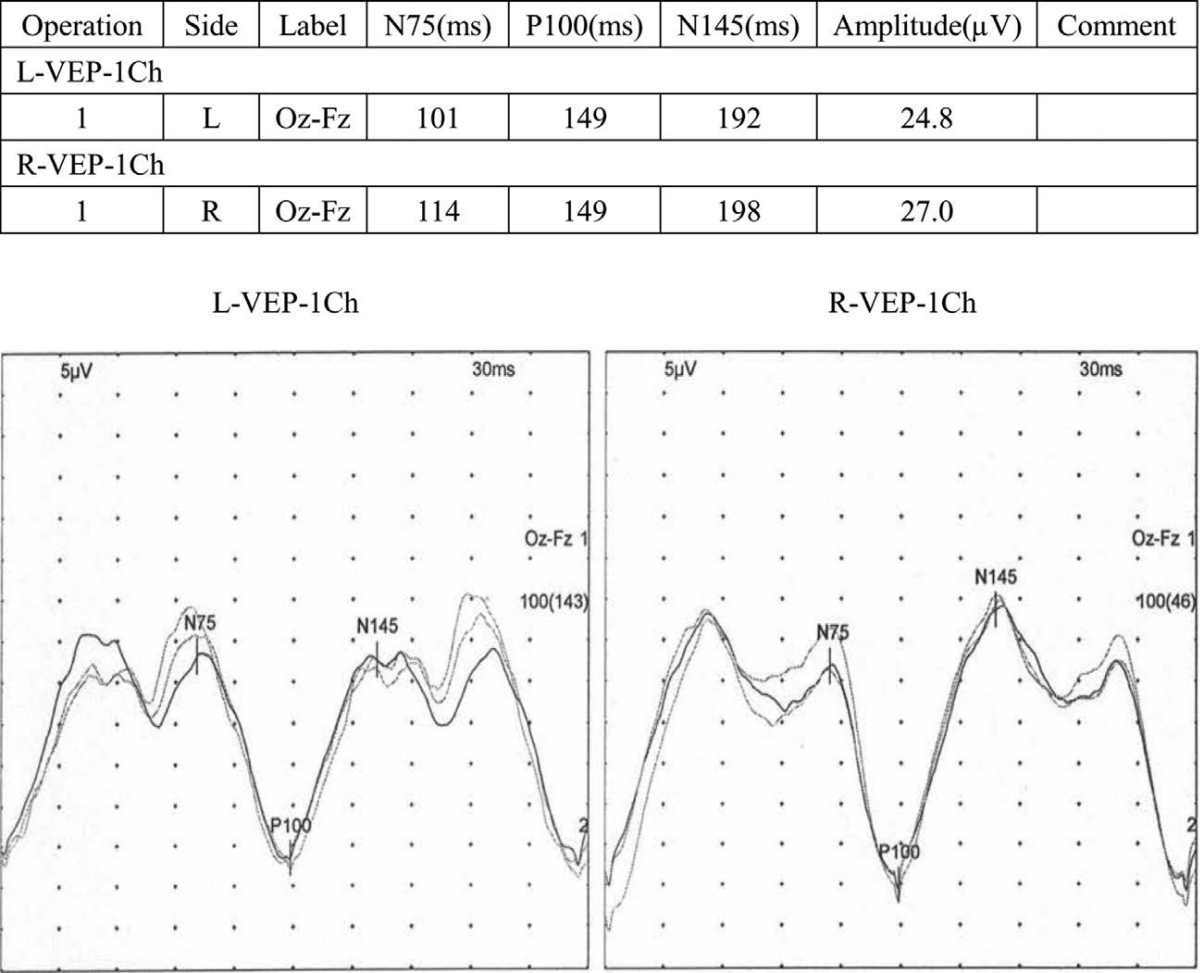
VEP: incubation period of bilateral P100 Prolonging. VEP = visual evoked potential.

Auxiliary examination, diagnosis, and treatment: The MRI of post-operation and this admission were shown in Figure 3. Compared with post-operation, the brain stem and cerebellum developed diffuse hyperintensity at this admission. The clinical symptoms and MRI are unable to provide sufficient evidence for us to differentiate astrocytoma recurrence from MOG-EM. Therefore, it is necessary for further examination. Due to the financial situation and the patient’s willingness, the patient and his family refused to undergo lumbar puncture and other traumatic operations except blood sampling. The serum antibody of MOG-EM was detected by the cell-based assay (CBA). The MOG-IgG was detected, and the titer ratio is 1 to 30. The outcome of serum antibody was presented in Figure 4. VEP was presented in Figure 5 which displayed incubation period of bilateral P100 Prolonging. As the above, we considered that the diagnosis of this patient was MOG-EM. With the consent of the patient and his family, the patients were given high-dose methylprednisolone intravenous injection (1000 mg/day × 3 days → 500 mg/day × 3 days → 240 mg/day × 3 days→120 mg/day × 3 days). The dizziness, vomiting, and vision loss were in remission after treatment of high-dose methylprednisolone. The patient received treatment of oral prednisone (60 mg/day) on discharge from hospital. Then 10 mg of prednisone was reduced every 7 days.

### 2.3. Follow-up after discharge

**Figure 6.**
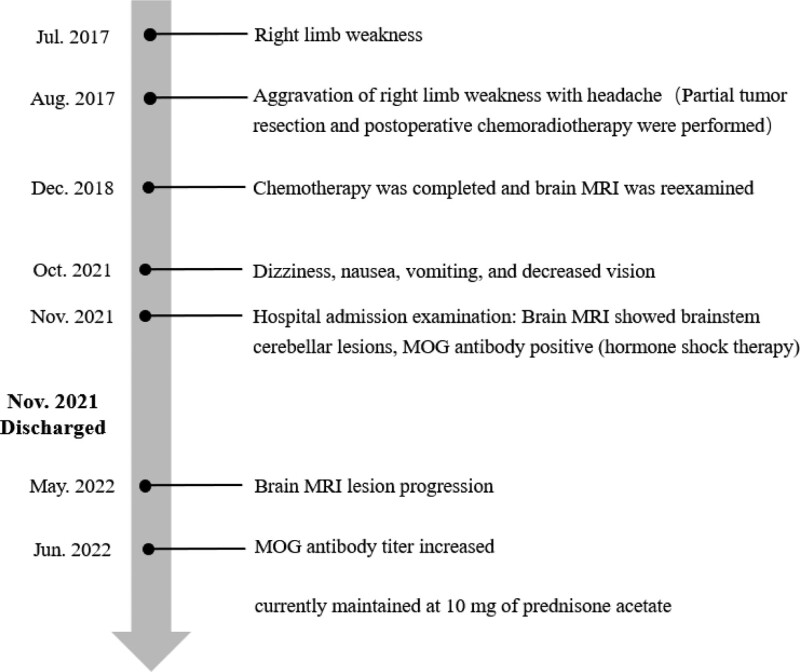
The summary of patient’s disease progression.

The patient was followed up to 7 months after discharge (June 29, 2022), and the patient did not experience any recurrence of symptoms. The brain MRI and serum antibody were used as items of reexamination. But MRI of reexamination displayed that the brain stem and cerebellum developed more diffuse hyperintensity than before (Fig. 3). The T1 of MRI was presented in Figure S1, Supplemental Digital Content, http://links.lww.com/MD/H532. The mass effect of brain stem and cerebellar lesions was more obvious during follow-up. And the titer of MOG-IgG was higher than before (Fig. 4). The titer ratio was 1 to 320 which went up more than 10 times. The outcomes of these examinations had finally confirmed the diagnosis of MOG-EM. The patient’s symptoms were relieved by oral prednisone after discharge until June 29, 2022, which has now been reduced to 10 mg orally per day. However, this did not stop the progression of the disease, and further treatment with azathioprine will follow, and the patient is still being followed up in the outpatient clinic. Figure 6 summarizes the patient’s disease progression.

## 3. Discussion

To the best of our knowledge, there are few studies and pathological reports on the correlation of MOG-EM and astrocytoma. The case of our report is rare because the patient has suffered from both astrocytoma and MOG-EM. There are relatively complete clinical cases preserved as well as auxiliary examination except surgical biopsy. As the patient underwent tumor resection at another hospital, the surgical biopsy examination was only preserved in the form of mobile phone photos. Fortunately, all evidence for the diagnosis of MOG-EM has been preserved completely. Therefore, the diagnosis of MOG-EM and astrocytoma is clear. However, the correlation between MOG-EM and astrocytoma remains unclear.

MOG-EM is a demyelinating disease of the central system with autoimmune disorder which is initially defined as disease about MOG-IgG-positive NMOSDs with the similar diagnostic criteria.^[1,8]^ As the development of acknowledge in MOG-EM, it should be diagnosed as a separate disease category. And the MOG-IgG has been detected in about 40% of the patients of NMOSDs without the AQP4-IgG which might be classified as MOG-EM.^[1,9,10]^ In previous studies, no significant agitating or inducing diseases associated with demyelinating MOG antibodies have been found. And MOG-EM is not usually explored in paraneoplastic syndrome. But this patient of our report developed MOG-EM after the astrocytoma. The etiology of this patient may have been speculated in previous MOG antibody studies. MOG is a type of glycoprotein on oligodendrocyte which is only found in central nervous system.^[11]^ And impairment of the blood brain barrier (BBB) may play an important role in pathogenesis of MOG-EM.^[12–14]^ The tumor itself might represent the immune response and immune monitoring abnormalities in human beings.^[6]^ In our case report, the impairment of BBB and oligodendrocyte might consist of four factors, including the impairment of tumor, tumor resection, postoperative radiation treatment and chemotherapy. These factors might lead to MOG-IgG positive which might play an important role in development of MOG-EM. The patient of our report is partly consistent with another case report.^[7]^ Therefore, we hypothesized that the onset of MOG-EM in this patient was related to astroglioma or its treatment.

The limitations of our study can be listed as follows: this study is a case report, which provides low level evidence based on evidence-based medicine; the follow-up time of this admission (about 7 months) is short, and new symptoms may occur in the future.

## 4. Conclusion

MOG-EM might be associated with astrocytoma or its treatment. What’s more, MOG-EM might be secondary to astrocytoma or its treatment. Astrocytoma should be considered as a high-risk factor for MOG-EM. More studies are needed to support and explore our findings.

## Author contributions

**Methodology:** Shaoming Yang, Hongli Gu.

**Writing – original draft:** Guanghong Zhong, Jia Zhang.

**Writing – review & editing:** Xi Liu.

## Supplementary Material


