# Supplementary material for: Astrocytoma with myelin oligodendrocyte glycoprotein antibody associated encephalomyelitis: A case report
Source: Medicine (Baltimore). 2022 Oct 7;101(40):e31003. doi: 10.1097/MD.0000000000031003 (PMC9542675; doi:10.1097/MD.0000000000031003)

Supplemental file 1: The T1 sequence of MRI

1. This admission (A) and follow-up after discharge (B);

2. Compared with this admission, the mass effect of brain stem and cerebellar lesions was more obvious during follow-up.

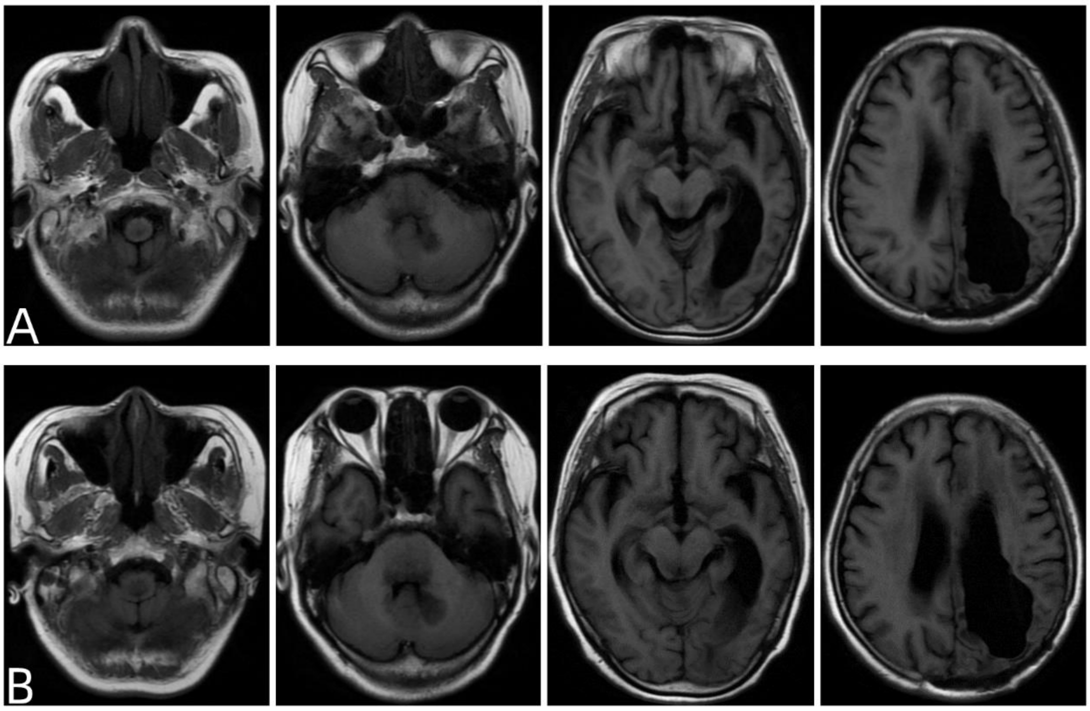

Supplement: Supplementary file 1 [file medi-101-e31003-s001.pdf]
